# Patient–Provider Communication and Counseling about Gestational Weight Gain and Physical Activity: A Qualitative Study of the Perceptions and Experiences of Latinas Pregnant with their First Child

**DOI:** 10.3390/ijerph14111412

**Published:** 2017-11-18

**Authors:** Ana Cristina Lindsay, Sherrie F. Wallington, Mary L. Greaney, Marcia M. Tavares Machado, Gabriela P. De Andrade

**Affiliations:** 1 Department of Exercise and Health Sciences, College of Nursing and Health Sciences, University of Massachusetts Boston, MA 02125, USA; gabriela.andrade001@umb.edu; 2 Department of Nutrition, Harvard T. H. Chan School of Public Health, Boston, MA 02115, USA; 3 Lombardi Comprehensive Cancer Center, Georgetown University Medical Center, Washington, DC 20057, USA; slw49@georgetown.edu; 4 Health Studies and Department of Kinesiology, University of Rhode Island, Kingston, RI 02881, USA; mgreaney@uri.edu; 5 Department of Community Health, Federal University of Ceará, Fortaleza, Ceará, 62010-560, Brazil; marciamachadoufc@gmail.com

**Keywords:** communication, patient–provider, gestational weight gain, physical activity, Latina, Brazilian

## Abstract

Latina women in the United States (U.S.) are disproportionately affected by obesity and are more likely to begin pregnancy overweight and gain excessive weight during pregnancy. The prenatal care period represents a window of opportunity for women to access the healthcare system and receive preventive services, education, nutritional support, and other social services to improve pregnancy outcomes. Excessive gestational weight gain (GWG) has numerous negative short- and long-term consequences for both the mother and newborn. We explored nulliparous Latina women’s perceptions about their experiences communicating with their primary healthcare provider about GWG and physical activity (PA) to identify possible intervention targets using in-depth, semi-structured interviews. Bilingual, trained research staff conducted 23 interviews with first-time pregnant Latinas between 22 and 36 weeks of gestation. Interviews were transcribed verbatim and analyzed using content analysis. Salient text passages were extracted, shortened, coded, and grouped into categories. Women, including those who self-identified as being overweight or obese prior to pregnancy, reported receiving limited or no advice from their healthcare providers about GWG or PA. Additionally, analysis revealed that although participants value information received from the Special Supplemental Nutrition Program for Women, Infants, and Children (WIC) program counselors, they would like to receive more information from their primary healthcare providers about adequate GWG. Furthermore, study findings indicate that some participants received conflicting information regarding PA during pregnancy. Study findings suggest the need for increased integration of communication and counseling about GWG and PA into prenatal care services to promote healthy weight gain and PA among low-income Latina women.

## 1. Introduction

Latinos are the largest and fastest growing minority group in the United States (U.S.) [1]. Latinas of childbearing age are at elevated risk of being obese (42.5% Latina/Hispanic vs. 32.6% non-Hispanic) and of beginning pregnancy with an overweight (body mass index (BMI) 25.0–29.9 kg/m^2^) or obese status (BMI ≥ 30.0 kg/m^2^) as indicated by pre-pregnancy BMI [2,3,4]. Preventing and reducing obesity among Latinas of childbearing age is an important public health goal and may contribute to closing disparities in obesity rates [2,4].

Excessive gestational weight gain (GWG) is common among Latinas [5,6,7], placing many at increased risk for pregnancy-related chronic disease [2,4]. Excessive GWG also increases the risk of adverse neonatal outcomes [8,9]. The Institute of Medicine (IOM) guidelines recommend that women with normal pre-pregnancy weight status gain 25–35 pounds during pregnancy, while women who are overweight or obese prior to pregnancy are advised to gain less weight (15–25 and 11–20 pounds, respectively) [10]. Prior research indicates that 36–51% of pregnant Latinas exceed the IOM guidelines for GWG [3,5,6].

It is recommended that pregnant women who are not already highly active or doing vigorous-intensity physical activity (PA) engage in at least 150 min of moderate intensity aerobic activity a week during pregnancy [10,11,12]. Healthcare providers should address PA when meeting with pregnant women because PA during pregnancy can aid in reducing excessive GWG and prevent postpartum weight retention [12,13,14]. Unfortunately, most pregnant women do not receive PA advice from their healthcare providers [15,16].

The prenatal care period provides an opportunity for women to receive preventive services, education, nutritional support, and other social services to improve pregnancy outcomes [2,8,9]. Patient–provider communication about GWG that addresses PA is central to effective prenatal care [10,11]. This is especially important for low-income, ethnic minority population groups such as Latinas, who are more likely to begin pregnancy overweight or with obesity [2,3,4] and may also have limited familiarity with GWG and PA guidelines, thus placing them at increased risk for excessive GWG [17,18]. The IOM recommends that women also receive counseling about GWG and PA in the prenatal care period [10], but between one-third and one-half of women do not receive counseling at all [15,16,17,18,19].

To date, there is still a dearth of qualitative studies examining Latina women’s perceptions of and experience with patient–provider communication and advice about GWG and PA. Prior qualitative research [20,21,22] suggests that Latina pregnant women receive minimal to no advice from healthcare providers related to GWG and PA recommendations. Moreover, to our knowledge, available studies have been limited to Spanish-speaking Latinas [3,7,12,13,20,21,22], and no studies have focused on Portuguese-speaking Latinas (Brazilians), a growing U.S. population group [23].

Understanding women’s perceptions of and experiences with patient–provider communication and advice about GWG and PA is important for the design of prenatal education and counseling interventions to promote healthy weight gain during pregnancy and ultimately reduce the risk of pregnancy-related chronic diseases. The purpose of this qualitative study was to explore the perceptions and experiences of Spanish- and Portuguese-speaking Latina women pregnant with their first child with respect to patient–provider communication and counseling about GWG and PA during pregnancy.

## 2. Materials and Methods

### 2.1. Study Design, Setting and Sample

The present study was part of community-based, mixed-method formative research being conducted (2015–present) with Latino families (multi-ethnic Hispanics and Brazilians) living in Massachusetts (MA) and Rhode Island (RI), designed to identify and explore factors influencing the risk of obesity in the first 1000 days of a child’s life [23,24,25].

This qualitative study used in-depth, semi-structured interviews with nulliparous pregnant Latina women to explore: (1) perceptions about GWG and PA during pregnancy; and (2) experiences with patient–provider communication related to GWG and PA during pregnancy. Given the dearth of information on this topic, qualitative methods were chosen to explore topic areas without predetermined quantitative questions [26]. In-depth, semi-structured interviews were the qualitative method chosen, since the open-ended nature of the questions not only defines the topics under investigation, but also provides opportunities for both interviewer and interviewee to discuss some topics in more detail [26]. In addition, during the in-depth interview, the interviewer has the freedom to probe the interviewee to elaborate on the original response or to follow a line of inquiry introduced by the interviewee that was not conceived a priori [26]. Furthermore, in-depth, semi-structured interviews were preferred for the current study over focus groups to ensure high participation rates. Given participants’ conflicting schedules it would have been more challenging to gather a group of pregnant women who were mostly in their third trimesters.

A convenience sample of participants was recruited through flyers posted at community-based and social programs, local agencies, and churches serving predominantly Latino populations (Hispanics and Brazilians) in MA and RI between April 2016 and February 2017. Interested women were screened by telephone to determine if they met the eligibility criteria. Eligible women were offered the option of participating in an interview at a place of their convenience. Prior to enrolling in the study, eligible women were read the consent forms in their preferred language (English or Spanish) by trained bilingual, bicultural research staff. Eligibility criteria included: (1) self-identifying as Latina (either Hispanic or Brazilian); (2) being nulliparous; (3) singleton pregnancy; (4) being at between 22 and 36 weeks of gestation; (5) being 18 years of age or older; (6) participating in or eligible for the Special Supplemental Nutrition Program for Women, Infants, and Children (WIC) program; (7) living in MA or RI; (8) residing in the US for at least 12 months; and (9) providing informed consent.

We chose to recruit participants from the WIC program because of its unique role in providing services to a large population of low-income mothers and children of ethnic minority backgrounds, including Hispanics and Brazilians. The WIC program is a food and nutrition education program for pregnant, breastfeeding, and postpartum women, infants, and children under age five who are low-income (up to 185% of the Federal Poverty Level) and at nutritional risk. The study protocol was approved by the Institutional Review Board (IRB#2013132) of the University of Massachusetts, Boston.

### 2.2. Data Collection

Two native speakers (one Spanish and one Portuguese-Brazilian) trained in qualitative research methods conducted all interviews in Spanish or Portuguese using a semi-structured interview guide with open-ended questions and probes. Using the socio-ecological model as framework, the pilot-tested interview guide was adapted from a prior study [27] and developed to explore two main topic areas: (1) participants’ perceptions and beliefs about GWG and PA during pregnancy; and (2) participants’ perceptions and experiences concerning the provider’s communication and advice about GWG and PA during pregnancy.

Interviews were held at public locations (library, community agency, church) convenient to the participant or, if requested, at the participant’s house. Interviews, which lasted approximately 45 min, were audiotaped after participants provided signed and oral informed consent. Before each interview, the interviewer explained procedures. All participants completed a brief, self-administered survey after the interview ended. The survey assessed participants’ socio-demographics (e.g., education, marital status, country of origin, length of time living in the US, etc.), self-reported weight status prior to start of pregnancy, and level of acculturation via the Short Acculturation Scale for Hispanics (SASH) [28]. The SASH is a 12-item measuring scale validated for use in Latino groups (e.g., Mexican Americans, Cuban Americans, Puerto Ricans, Dominicans, and Central and South Americans) that assesses language use, media use, and ethnic social relations. An acculturation score was computed by averaging across 12 items as measured on a scale of one to five (1 = least acculturated, 5 = fully acculturated) [28]. Participants received a $25 gift card for their participation in the study.

### 2.3. Data Analysis

Audiotapes were transcribed verbatim in Spanish or Portuguese and translated into English without identifiers. To ensure that the integrity and equivalence of the data were not lost during translation, professional transcriptionists who are bilingual and native Spanish and Portuguese speakers translated the transcripts using forward–backward techniques to establish semantic equivalence in translation [29]. Transcripts were analyzed using thematic analysis, an iterative process of coding data in phases to create meaningful patterns [29]. Analytic phases included data familiarization, generation of initial codes, identification and review of themes, and defining and naming themes [30]. Two authors (ACL, MMTM), both experienced qualitative researchers, independently conducted all analyses, checked for consistency between their analyses, and resolved discrepancies. Disagreements were discussed until a consensus was reached. Descriptive statistics were calculated for the socio-demographic data using Microsoft Excel 2008^®^ (Redmond, WA, USA).

## 3. Results

Twenty-three interviews were conducted with a diverse sample of Latinas (12 multi-ethnic Hispanics and 11 Brazilians) before saturation was reached. As seen in Table 1, participants were between 22 and 35 (mean = 24.1, SD = 2.3) years of age, low-income, nearly all foreign-born, had lived in the US for an average of 8.2 years (SD = 2.4), and all spoke Spanish or Portuguese. Participants had a mean acculturation score of 1.62 (SD = 0.37), indicating that they identified more closely with the Latino culture than with the American culture [28]. Moreover, a little over half (53.2%, *n* = 12) of the participants reported being of normal weight, while about 44% (*n* = 10) reported being overweight, and only one participant self-reported having obesity prior to the start of pregnancy.

Analysis identified nine emergent themes. Themes with illustrative quotes are presented below in two broad categories: (1) patient–provider communication and advice about GWG; and (2) patient–provider communication and advice about PA during pregnancy.

### 3.1. Patient–Provider Communication and Advice about GWG

Six main themes emerged related to patient–provider communication and advice about GWG, all of which are discussed below.

#### 3.1.1. Theme 1: Women Received Limited Advice from Their Primary Healthcare Provider about GWG

The majority of participants reported that their primary healthcare providers tracked their weight gain during pregnancy, but did not provide them with specific information as to whether their weight gain was appropriate.

“Every visit the nurse assistant weighs me, and the doctor checks it in my chart, but he [physician] never really tells me much about it—just that it [weight] is okay. One visits the nurse assistant told me: ‘Wow, you gained 6 pounds!’ But then the doctor didn’t say anything about my weight during the visit, so I figured it was probably okay.”

—Dominican #1, self-reported as overweight before pregnancy

The few women who reported receiving minimal information about GWG stated that their providers let them know that their weight gain was within the normal range, but no further information on specific healthy weight ranges or future weight gain goals/expectations were discussed.

“My doctor doesn’t really talk much about my weight gain. She mentioned it (weight gain) briefly during my first prenatal care visit… But, she always looks at the chart and tells me that I am doing well and that my weight gain is normal for the third trimester. I do feel I am gaining more weight now.”

—Colombian#1, self-reported as normal weight before pregnancy

#### 3.1.2. Theme 2: Women Perceived Their Primary Healthcare Providers to be Unconcerned about GWG

Women felt that their providers were not concerned about their GWG.

“I am 37 weeks pregnant, and I don’t really remember my doctor ever talking to me about my [gestational] weight gain. He looks at the chart that the nurse gives him and says all looks fine. So, I don’t think he’s really worried about my weight gain. I feel huge, but I think I am doing fine. This is my first baby, so it’s hard for me to know if my weight gain is normal or not.”

—Brazilian #1, self-reported as normal weight before pregnancy

“I feel like I am gaining a lot more weight now (33 weeks pregnant), but my doctor never really say anything about my weight gain being bad. I don’t ask either. If he doesn’t say anything, I don’t feel like asking. Sometimes when I go to the WIC clinic, I ask the nutritionist and she explains it to me.”

—Salvadoran #1, self-reported as overweight before pregnancy

Some women who reported being overweight or having obesity pre-pregnancy felt that their primary healthcare providers were not concerned about their GWG. They believed that if their weight gain was concerning, their provider would discuss it with them. In addition, some women who perceived their pregnancy weight gain as being excessive reported that as long as the baby was developing normally, the weight gain was not as important and could wait to be addressed after the baby was born.

“My doctor does not talk much about my weight gain. I was already overweight before getting pregnant, so I think she (the physician) know that I am aware of my weight not being where it should be. If she doesn’t mention, I am not going to ask it. I don’t feel like asking it. As long as the baby is fine, I am fine.”

—Puerto Rican #3, self-reported as obese before pregnancy

#### 3.1.3. Theme 3: Reliance on Multiple Sources of Information about GWG

The majority of women reported getting information from the internet, books, and interpersonal communication with family member and friends who had children and were therefore perceived as being knowledgeable.

“This is my first pregnancy, so I don’t have any experience, but I am curious and want to know as much as possible to make sure I have a healthy pregnancy and baby. So, I am always searching for information in the internet and reading magazines, and any information I come across.”

—Brazilian #4, self-reported as overweight before pregnancy

“This is my first baby, so I talk a lot with my mom, my aunties, and my friends who have children. I feel that they at least know some from their experience with being pregnant.”

—Dominican #2, self-reported as overweight before pregnancy

#### 3.1.4. Theme 4: WIC Program and Staff Play an Important Role in Providing Information about GWG

Most women reported receiving information about GWG from WIC nutritionists during their WIC visits, and that they appreciated receiving this information.

“When I go for my WIC visits, the WIC nutritionists always talk to me about my weight gain. She tells me if I am doing well, if everything is good. Sometimes she tells me to watch what I am eating and to make sure I don’t gain too much weight.”

—Salvadoran #1, self-reported as overweight before pregnancy

“Whenever I have questions I ask the WIC nutritionist. She is very good at explaining things and I feel comfortable with her.”

—Dominican #3, self-reported as overweight before pregnancy

#### 3.1.5. Theme 5: Women Encounter Language Barriers that Impact Patient-Provider Communication

The majority of women reported having access to and using a professional interpreter during their prenatal care visits.

“I am not fluent in English, so I always prefer to have an interpreter during the visit, unless a friend or a family member who speaks English fluently accompanies me.”

—Brazilian #10, self-reported as normal weight before pregnancy

Several women, however, felt that having an interpreter present as a third party during their prenatal care visits prevented them from being able to express themselves freely and to communicate directly with their providers. A few women indicated that they felt uncomfortable asking their provider multiple questions about their weight gain through an interpreter.

“I am thankful that the hospital has an interpreter to help me during my visit, but it’s not the same as talking with someone in your own language. It’s like you are talking with one person and not knowing if what you are saying is really being understood …sometimes it’s easy to get lost. But if you don’t speak the language, that’s the way it is.”

—Brazilian #5, self-reported as normal weight before pregnancy

“I feel strange asking the doctor questions about my weight through the interpreter. I don’t know, I just don’t feel comfortable. So, if my doctor doesn’t mention anything (about weight gain), I just don’t ask.”

—Puerto Rican #2, self-reported as overweight before pregnancy

#### 3.1.6. Theme 6: Participants Want More Information about GWG from Their Primary Healthcare Providers

Overall, the majority of women reported that they would like to receive more information and advice about GWG from their physicians.

“I wish that right from the start she (the physician) would tell me how much weight I could gain and explain how I could achieve that, but she (the physician) never really talked about it, and I did not feel like asking it.”

—Puerto Rican #3, self-reported as obese before pregnancy

Moreover, a few women reported that although they liked getting advice from the WIC nutritionist they would have preferred to receive this information from their primary healthcare providers.

*“I was happy that the nutritionist at the WIC clinic always told me whether I was doing well or not with my weight gain. I trust her, but I think I would have felt better if my doctor also told me I was doing well. I don’t know, but one always think that the doctor knows more.”*

—Puerto Rican #4, self-reported as overweight before pregnancy

### 3.2. Patient–Provider Communication and Advice about PA during Pregnancy

The three themes that emerged related to PA and patient–provider communication about PA during pregnancy are presented below.

#### 3.2.1. Theme 1: Participants’ PA Declined during Pregnancy

Although most women reported being physically active before getting pregnant, only a few reported maintaining the same physical activity levels during pregnancy. Women who reported being physically active during their pregnancy said their activity was limited to daily household or job-related chores. Women spoke of “not having energy”, “feeling tired all the time” or being concerned that being physically active could be “harmful to the baby” as the main reasons for their limited PA. In addition, a few women explained that they had a difficult first trimester with morning sickness, which prevented them from being physically active. Furthermore, several women reported being concerned that they would never return to their pre-pregnancy level of activity.

“Most of the exercise I do is cleaning the house and standing at work. I am really tired all the time, and I don’t feel like doing much.”

—Dominican #4, self-reported as normal weight before pregnancy

“I used to be very physically active before getting pregnant. I was always walking everywhere, going to the gym, but after I got pregnant, I just did not have much energy. All I feel like doing is lying down. I wonder if I will ever be able to be as physically active as I was before getting pregnant.”

—Brazilian #8, self-reported as normal weight before pregnancy

#### 3.2.2. Theme 2: Women Received Limited or No Advice about PA from Their Providers

Overall, most women reported receiving limited or no advice about PA from their primary healthcare providers.

“My doctor never mentioned physical activity or exercise; it just never came up during my visits, and I never felt I needed to ask either.”

—Guatemalan woman #1, self-reported as overweight before pregnancy

The few women who reported receiving PA advice felt that their provider’s advice conflicted. For example, one participant mentioned that her provider discussed information related to the importance of PA during pregnancy for maintaining a healthy weight but also told her to be cautious about the types of physical activities. Similarly, a couple of women reported receiving advice to avoid extraneous PA and/or to be cautious and limit their exercise during pregnancy.

“My doctor only mentioned physical activity and exercise in my first appointment. He talked about a lot of things about pregnancy. I think because this is my first pregnancy …What I really remember is that he told me to be careful with doing too much physical activity and what sorts of activities …that’s what I remember. I don’t really exercise that much. Mainly doing things around the house …”

—Puerto Rican #2, self-reported as overweight before pregnancy

“My doctor and also the nurse told me I should be careful about exercising now that I was pregnant. They said, ‘Don’t do anything that’s too hard on your body and dangerous for the baby.’”

—Puerto Rican #1, self-reported as overweight before pregnancy

#### 3.2.3. Theme 3: Women Seek Information about PA from the Internet as well as Family and Friends

Overall, the majority of women reported that their primary healthcare providers did not provide them with information or advice about PA, and that they relied on other sources for information. Women who had questions about PA or exercising during pregnancy turned to the Internet or relied on interpersonal communication with family members and friends who had been pregnant before for information.

“If I had any questions about whether or not I should exercise, I would check on the Internet. I felt I was able to get the information I needed. I also talked with my friends and relatives. Some had been pregnant before, so I felt that their advice was helpful.”

—Brazilian #6, self-reported as normal weight before pregnancy

“My health care provider never really talked to me about exercise, so whenever I had any questions I would ask a friend or my cousin who had been pregnant before, or I would also look online.”

—Dominican #4, self-reported as normal weight before pregnancy

## 4. Discussion

This study provides an overview of nulliparous, low-income Latina women’s perceptions of their experiences with patient-provider communication and advice about GWG and PA. The most important finding of this study was that participants, including women who self-identified as being overweight prior to pregnancy, reported a lack of or limited advice from their healthcare providers on GWG. This finding is consistent with prior research on women from multiple racial/ethnic and socioeconomic backgrounds [16,19,31,32,33]. In addition, women in the current study reported that this lack of communication and counseling about GWG left them unsure as to whether their GWG was appropriate or concerning. Consistent with prior research, study findings showed that women wanted more information from their healthcare providers on GWG and that they especially valued receiving advice from their primary healthcare providers [27,32,33]. A provider’s advice is critical in helping women meet appropriate GWG targets as set by the IOM [10,11], and it may be even more critical for racial/ethnic minority women who may be less aware of GWG recommendations [11] and more likely to exceed recommended GWG [3,6,22].

Another notable study finding is that women received very limited to no advice from their primary healthcare providers about being physically active during pregnancy. This lack of information is concerning since PA during pregnancy can limit excessive GWG and prevent postpartum weight retention [34,35]. Taken together, study findings highlight the need for increased communication on the part of primary healthcare providers about weight status and PA during pregnancy. There is a need for interventions targeting both providers and patients about the importance of communication about GWG and PA to address this communication gap. In addition, study findings suggest that interventions targeting Latina pregnant women should consider targeting the development of skills and self-efficacy to communicate with providers about GWG and PA.

A novel study finding is that most participants reported receiving advice on GWG from the WIC program. It is important to note that the primary healthcare providers of women participating in this study may have known that their patients are seen by WIC staff (e.g., nutritionists), and may have relied on these healthcare professionals to provide advice and, as a result, may not have counseled their patients about GWG due to time constraints. Nonetheless, this study finding suggests the potential role the WIC program and its staff can play in delivering prenatal nutritional services to address educational needs of Latina women about GWG.

The lack of patient–provider communication about GWG and PA led most women to seek information from other sources such as the Internet, books, and interpersonal communication with family members and friends with children. Our findings concur with previous research [8,22,33] that demonstrates that women need access to accurate and reliable sources of information to achieve healthy outcomes during the prenatal and postnatal periods [8,11,17]. Understanding Latina women’s beliefs and experiences with GWG and PA during pregnancy is critical to the development of interventions that seek to optimize recommended GWG [17]. Additional research should explore these topics as well as providers’ beliefs and practices related to GWG and PA counseling for low-income, Latina pregnant women.

Findings of this study revealed that for women who relied on interpreters felt that this reliance was a barrier to their communication with their primary healthcare providers and kept them from engaging in more communication with their providers about their GWG. Similar to our previous research with immigrant women of reproductive age, women in the current study felt that not being unable to communicate directly with healthcare professionals was frustrating and that this contributed to reduced communication and interactions [23]. Our findings concur with previous research showing that although the use of a professional interpreter for encounters with patients with limited English skills improves clinical care, patients’ satisfaction and outcomes with care received are diminished [36,37,38]. Study findings suggest that additional attention by healthcare providers should be given to minority women with limited language proficiency whom are at increased risk of excessive GWG such as Spanish- and Portuguese speaking pregnant women participating in our study. Making interactive, low-literacy, educational materials available in Spanish and Portuguese could help facilitate communication of important information (e.g., guidelines) to pregnant women with limited English proficiency. In addition, healthcare providers could build on information provided by such materials to further assess women’s understanding of guidelines and recommendations related to GWG and PA.

Understanding women’s perceptions of and experiences with patient–provider communication and advice about GWG and PA has important implications for the design of prenatal education and counseling interventions [17]. This information is especially important for low-income Latina women who are at increased risk of inadequate GWG and related short- and long-term consequences for both baby and mother [4,17,18]. Study findings suggest the importance of integration of communication and counseling about GWG and PA into prenatal care services to meet the needs of low-income, minority Latina women.

Study results should be considered in light of some limitations. Findings are based on a nonrandom, purposive, and relatively small sample of low-income Latina women in selected communities in MA and RI. Furthermore, the women who participated in this study may have been more aware of and concerned with issues related to patient–provider communication and advice about GWG and PA. In addition, this study relied on self-reported information from pregnant women and not from providers, including the self-reported BMI category, which may lead to misclassification (underestimation), which may increase with increasing measured BMI [39]. Overweight or obese women may be more likely to misclassify their weight status than women of normal weight [39]. Future research can address these limitations by examining patient–provider communication and advice from both multi-ethnic Latina women and providers from these and other communities across the US. In addition, quantitative research that builds on the qualitative findings reported in the present study is needed to quantify patient–provider communication and counseling about GWG and PA from both Latina pregnant women and providers.

## 5. Conclusions

Central to effective prenatal care is patient–provider communication about GWG and PA guidelines during pregnancy [10,11]. This is especially important for low-income, ethnic minority population groups who may have limited familiarity with these guidelines and may be at increased risk for excessive GWG. Our findings suggest that there is a need for increased communication and counseling between primary healthcare providers and Latinas about GWG and PA to reduce disparities in maternal obesity and improve the health of newborn children. Given that racial/ethnic differences are non-modifiable risk factors for excessive gestational weight gain, the prenatal and postnatal care periods represent critical windows of opportunity for obesity and chronic disease prevention among Latina women and their children.

## Figures and Tables

**Table 1:** Socio-demographic and acculturation characteristics and self-reported weight status of study participants (*n* = 23).

Variables	Mean + SD	*n* (%)
**Age (Years)**	24 ± 2.3	
**Race**		
Brazilian		11 (47.8)
Hispanic		12 (53.2)
**% Foreign-Born**		22 (95.6)
**Country of Origin**		
Brazil		11 (47.8)
Dominican Republic		4 (17.3)
Puerto Rico (United States)		4 (17.3)
Colombia		2 (8.6)
El Salvador		1 (4.4)
Guatemala		1 (4.4)
Years in the United States *	8.2 ± 2.4	
**Predominant Language Spoken at Home**		
Spanish		12 (53.2)
Portuguese		11 (47.8)
**Marin Scale Acculturation Score**	1.62 ± 0.37	
**Marital Status**		
Married		19 (82.7)
Single		4 (17.3)
**Educational Level**		
Less than high school		6 (26.1)
High school degree		10 (43.6)
General Educational Development (GED)		4 (17.3)
Some college or more		3 (13.0)
**Household Annual Income**		
>$20 K/year <$40,000		19 (82.7)
<$20 K/year		4 (17.3)
**Percent Employed**		17 (73.9)
**Self-Reported BMI (kg/m^2^) Prior to Pregnancy**		
Normal		12 (53.2)
Overweight		10 (43.6)
Obese		1 (4.4)

* Limited to foreign-born women. (BMI ) Body Mass Index.

## Abbreviations

GWG	Gestational weight gain
PA	Physical activity
US	United States
IOM	Institute of medicine
SASH	Short acculturation scale for hispanics
WIC	Special supplemental nutrition program for women, infants and children
MA	Massachusetts
RI	Rhode Island
SD	Standard deviation
